# HER2 expression on tumor-derived extracellular vesicles and circulating tumor cells in metastatic breast cancer

**DOI:** 10.1186/s13058-020-01323-5

**Published:** 2020-08-12

**Authors:** Afroditi Nanou, Leonie Laura Zeune, Francois-Clement Bidard, Jean-Yves Pierga, Leonardus Wendelinus Mathias Marie Terstappen

**Affiliations:** 1 Department of Medical Cell BioPhysics, Faculty of Science and Technology, University of Twente, Carré Room CR4433, Hallenweg 23, 7522 NH Enschede, The Netherlands; 2 Department of Medical Oncology, Institut Curie, PSL Research University, Paris and Saint Cloud, France; 3 Circulating Tumor Biomarkers Laboratory, SiRIC, Institut Curie, PSL Research University, Paris, France; 4 UVSQ, Paris-Saclay University, Paris, France; 5 Université Paris Descartes, Paris, France; 6 Department of Medical Cell BioPhysics, Faculty of Science and Technology, University of Twente, Carré Room CR4437, Hallenweg 23, 7522 NH Enschede, The Netherlands

**Keywords:** Circulating tumor cells, Tumor-derived extracellular vesicles, Human epidermal growth factor receptor 2, HER2, CellSearch, ACCEPT

## Abstract

**Background:**

Tumor-derived extracellular vesicles (tdEVs) and circulating tumor cells (CTCs) in the blood of metastatic cancer patients associate with poor outcomes. In this study, we explored the human epidermal growth factor receptor 2 (HER2) expression on CTCs and tdEVs of metastatic breast cancer patients.

**Methods:**

Blood samples from 98 patients (CLCC-IC-2006-04 study) were originally processed with the CellSearch® system using the CTC kit and anti-HER2 as an additional marker in the staining cocktail. CTCs and tdEVs were automatically enumerated from the generated CellSearch images using the open-source ACCEPT software.

**Results:**

CTCs and tdEVs were subdivided based on their cytokeratin (CK) and HER2 phenotype into CK+HER2−, CK−HER2+, and CK+HER2+. The inclusion of anti-HER2 increased the percentage of informative samples with ≥ 1 detectable CTC from 89 to 95%. CK− CTCs and tdEVs correlated equally well with the clinical outcome as CK+ CTCs and tdEVs. Inter- and intra-patient heterogeneity was found for the CTC/tdEV phenotypes, and the presence of 2 or 3 classes of CTCs/tdEVs was associated with worse prognosis compared to a uniform CTC/tdEV phenotype present (1 class). The use of ≥ 7% HER2+CK+ tdEVs can predict HER2 expression of the tissue with 74% sensitivity and specificity using the HER2 amplification status of the primary tumor as a classification variable.

**Conclusions:**

HER2 can be detected on CTCs and tdEVs not expressing CK, and these CK− CTCs/tdEVs have similar clinical relevance to CTCs and tdEVs expressing CK. tdEVs perform better than CTCs in predicting the HER2 status of the primary tissue. CTC and tdEV heterogeneity in the blood of patients is inversely associated with overall survival.

## Background

The availability of more and more targeted treatments necessitates the development of techniques to screen cancer patients for the presence of the respective therapeutic targets in the primary and metastatic lesions. However, the invasiveness of sampling of biopsies from all different metastatic lesions and the associated patient morbidity demands the need for a less invasive test. EpCAM+ circulating tumor cells (CTCs) isolated from the peripheral blood of cancer patients constitute clinically relevant and easily accessible tumor material from a single tube of blood [1, 2]. The possibility of longitudinal measurements and the screening of CTCs for therapeutic targets can provide clinicians with the mutational status of the tumor and the presence of treatment targets in real-time facilitating their decisions on treatment monitoring [3, 4]. The reliability of such an assessment increases with the number of CTCs available. In metastatic breast cancer, ~ 50% of patients have ≥ 5 CTCs detected with the CellSearch system and the percentage of patients with ≥ 10 and ≥ 100 CTCs decreases rapidly [1].

The development of the open-source ACCEPT software allowed the automated enumeration of all objects in the fluorescence images and the more objective assessment of treatment targets on CTCs [5] eliminating inter- and intra-operator variations [6].

Large tumor-derived extracellular vesicles (tdEVs) are co-isolated with CTCs and are present in an order of magnitude higher frequencies than CTCs [7, 8]; thus, they can increase the number of “readable” patient samples for the assessment of therapeutic targets.

The original study used in the present analysis is the CLCC-IC-2006-04 (NCT00898014) with 267 stage IV breast cancer patients receiving first-line chemotherapy [9]. The primary objective was to predict overall survival (OS) and progression-free survival (PFS) by counting all CK+ CTCs before the initiation of the second course of chemotherapy [9]. The secondary objective was to evaluate the HER2 status on CTCs over the course of a treatment. Ligthart et al. [3] and Zeune et al. [5] quantified the HER2 expression on the pre-scored by the operator CTCs in an automated manner demonstrating a more objective and less biased assessment among the different operators.

In the present study, we wanted to address two important questions:

i.) Is HER2 expressed on tdEVs?
ii.) Is HER2 expressed on CTCs lacking CK expression? And if so, do these populations associate with the clinical outcome of the patients?

To address the aforementioned research questions, the open-source ACCEPT software was used and the enumeration of all CTCs and tdEVs falling in the different immunophenotypic classes was performed in an automated manner.

## Methods

### CellSearch images of breast cancer patients

Digitally stored CellSearch® (Menarini Silicon Biosystems, Huntingdon Valley, PA, USA) image files from 98 metastatic breast cancer patients from a previously reported study (CLCC-IC-2006-04 study, ClinicalTrials.gov Identifier: NCT00898014) were re-analyzed [3, 9]. The original study included 267 patients with either HER2+ or HER2− primary tumor, whose characteristics at the baseline have been previously described [9]. Of these samples, 114 were further screened for HER2 expression on CTCs. These samples had been processed with the CellSearch system using the CTC kit, as previously described [7] including an additional fluorescein (FITC)-conjugated monoclonal antibody recognizing HER2 (clone Her81) in the staining mixture [11]. The specific clone recognizes an epitope in the extracellular domain of HER2, and it does not cross block binding of the FDA-cleared trastuzumab used in the clinics [11]. Ninety-eight out of 114 image datasets (where the anti-HER2 had been included in the staining mix) were available for the present analysis.

### Classification and automated enumeration of CTCs and tdEVs using ACCEPT

For the automated CTC and tdEV count enumeration, the digitally stored fluorescence image files were processed with the Automated CTC Classification, Enumeration and PhenoTyping (ACCEPT) software v1.1 (http://github.com/LeonieZ/ACCEPT) using the “Full Detection” function. The ACCEPT software detects all objects, present in the fluorescence images with a size larger than 4 pixels, and it extracts for each of them measurements of 10 morphological and fluorescence intensity features per fluorescence channel [12]. The operator can define the classes of their interest by designing linear gates using the desired features. Once optimized, the gates can be applied in all samples, and the counts of objects falling within them can be automatically extracted. The application of the same gates for all samples allows fast enumeration and elimination of inter- and intra-operator variability and bias leading to a more objective consensus [6].

The herein used gates took into account the roundness (eccentricity and perimeter to the area), min and max sizes (area and perimeter), mean fluorescence intensity of all different channels, and the overlap of CK/HER2 with DNA (in case of CTCs). The exact gates that were applied can be found in Supplementary Table S1, Additional file 3.

### Statistical analysis

Statistical analysis was performed in IBM SPSS Statistics v24.0 (SPSS Inc., Chicago, IL, USA) and MedCalc v19 (MedCalc Software, Ostend, Belgium). For the matched CTC and tdEV counts of the same subclass, a two-tailed Spearman’s rho test was performed to evaluate their relation through a monotonic function and the non-parametric Wilcoxon signed ranks test was used to test the equality of their distributions. The non-parametric Mann-Whitney *U* test was used to compare the distributions for the absolute and relative frequencies of CTCs and tdEVs with different immunophenotypes. The open-source web application Cutoff Finder (http://molpath.charite.de/cutoff/) was used to calculate the hazard ratios (HRs) for overall survival (OS) including 95% confidence intervals (CIs), over a wide range of cutoff values for CK+/− CTCs and tdEVs. Cutoff Finder uses the R code to provide optimization and visualization tools for cutoff determination [13]. Receiver operating characteristic (ROC) curves were used to evaluate the performance of %HER2+ CTCs (or tdEVs) in predicting HER2 amplification status of the primary tumor as assessed by FISH (available data for 92/98 patients). The areas under the curve (AUCs) were compared using the non-parametric DeLong approach [14]. We determined a minimum threshold for %HER2+CK+ CTCs or tdEVs to predict a HER2+ tissue as the value that led to equal sensitivity and specificity (value that led to minimum |sensitivity − specificity|). OS was defined as the elapsed time between blood draw and death, and it was available for 94/98 patients. The patients who were still alive at the last follow-up were censored. Kaplan-Meier (KM) survival curves of OS were used to compare (using the log-rank test) patients with favorable and unfavorable manual and automated CTC and tdEV counts. Cox proportional hazards regression analysis was used to determine the univariable HRs with 95% CIs of the categorical and continuous CTCs and tdEVs for OS. Continuous CTC and tdEV counts were log-transformed to achieve a better model fit.

## Results

### Patient subpopulation included in the present analysis

The patients included in the present study were not chosen based on their CTC counts; that would lead to a selection bias. There was no significant difference in regard to the probability distribution for OS (*p* = 0.863, log-rank test) and the distributions for the manual CTC counts (*p* = 0.600, Mann-Whitney *U* test) between the complete patient cohort and the patient subpopulation included in the present analysis. The Kaplan-Meier of OS for the complete patient cohort of the study and the patient subpopulation chosen as well as the box plots of the manual CTC counts of the corresponding patients are shown in panels A and B of Additional file 1, respectively.

### ACCEPT display and automated enumeration of CTCs and tdEVs

After the inclusion of the HER2-FITC antibody, 3 different subclasses of CTCs and tdEVs were detected in the CellSearch images (Additional file 2). Linear ACCEPT gates were designed (Supplementary Table S1, Additional file 3) and applied in the image datasets for the automated enumeration of CTCs/tdEVs of each subclass.

**Fig. 1:**
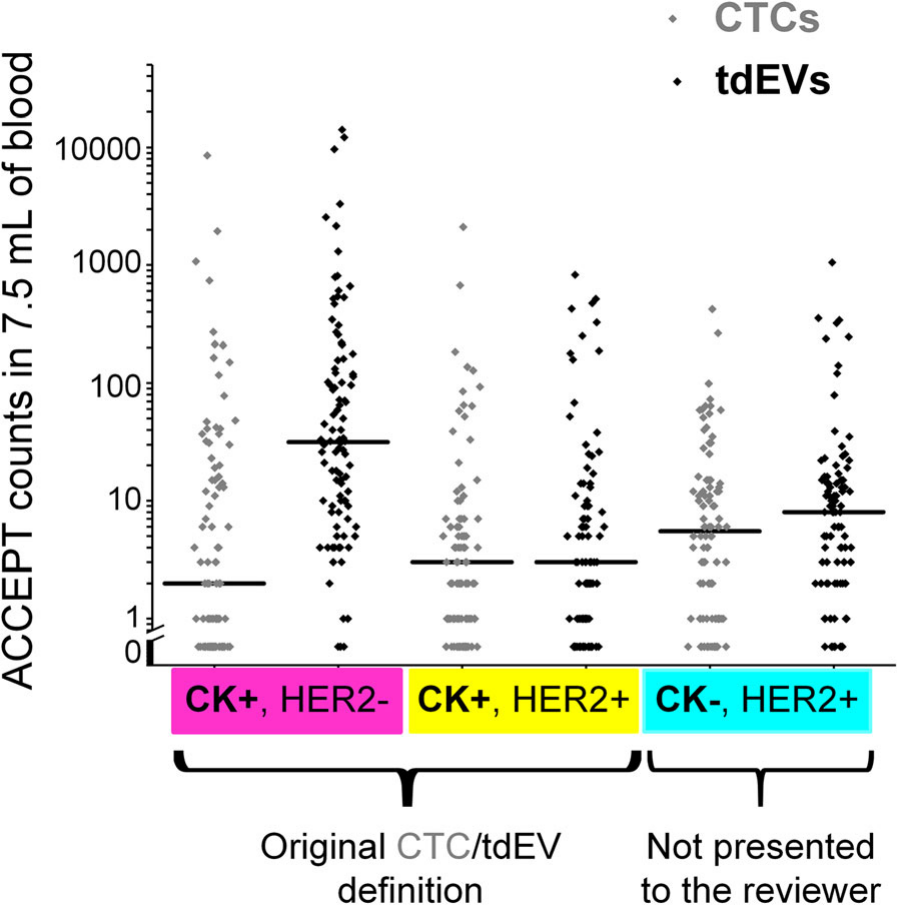
Frequencies of CTC and tdEV subpopulations in metastatic breast cancer patients. Dot plots of different subclasses (indicated in the *x*-axis) of CTCs (in gray) and tdEVs (in black) in 98 metastatic breast cancer patients. Each class/immunophenotype is indicated by a different color on the *x*-axis. Each dot corresponds to the counts of one patient. Horizontal black lines indicate the median values

The automated CTC and tdEV counts of the 98 breast cancer patients are shown in dot plots in Fig. 1. Comparison of the applied ACCEPT gates for CTC automated enumeration and the manual CK+ CTC counts scored by the operator in the original study showed a high correlation (*r*_S_ = 0.83, *p* < 0.01) and similar association to the clinical outcome (Additional file 4).

Ninety-three of 98 (95%) patients had ≥ 1 detectable CTCs, and 98/98 (100%) of patients had ≥ 1 tdEV (Fig. 1). Whereas CK+HER− tdEVs are found in a ~ 15-fold higher frequency compared to CK+HER− CTCs (*p* < 0.01, Wilcoxon signed ranks test), the distributions of CK+HER2+ and CK−HER2+ tdEVs are not significantly different compared to the respective distributions of CTCs (Wilcoxon signed ranks test). Furthermore, the correlation between CTC and tdEV subclasses was very strong only in case of CK+HER2− populations (*r*_S_ = 0.84, *p* < 0.01), whereas in case of CK−HER2+ and CK+HER2+ populations, a moderate correlation was found (0.5 < *r*_S_ < 0.6, *p* < 0.01).

### Association of CK+ and CK− CTCs and tdEVs with clinical outcome

**Fig. 2:**
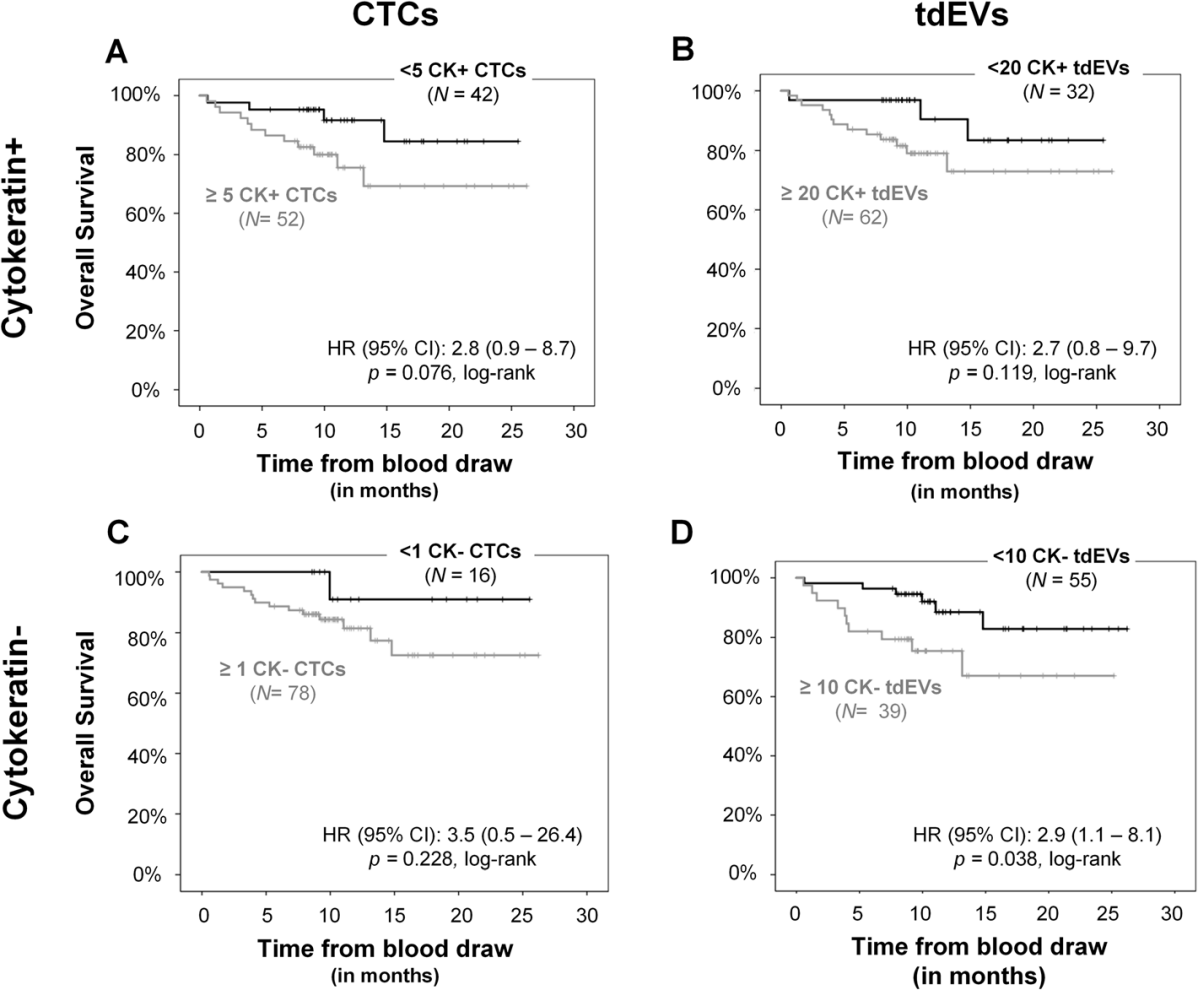
Association of CK+ and CK− CTCs and tdEVs with the clinical outcome of metastatic breast cancer patients. KM curves for OS dichotomizing patients based on their automated CK+ CTCs (**a**) and tdEVs (**b**) and their CK− CTCs (**c**) and tdEVs (**d**)

The original CellSearch CTC definition requires the expression of CK within the nucleated cells of a size larger than 4 μm, after EpCAM enrichment. Similarly, our previously reported tdEV definition enclosed all events isolated with the CellSearch system that were positive for CK and negative for CD45, without a nucleus and with a size range between 1 and 14 μm [8]. The CK- and CD45-negative cell and EV populations that are expressing a treatment target, such as HER2, could indicate tumorigenic origin. To determine whether these lacking CK and expressing HER2 objects are actually cancer-associated, we named them CK− CTCs and tdEVs and compared their prognostic power with CTCs and tdEVs expressing CK. For that comparison, KM plots for OS were generated (Fig. 2) stratifying patients based on their CK+ CTCs (panel a), CK+ tdEVs (panel b), CK− CTCs (panel c), or CK− tdEVs (panel d). The selected cutoffs were 5 for CK+ CTCs (established cutoff value), 1 for CK− CTCs (since we wanted to evaluate whereas the presence itself is associated with worse prognosis), 20 for CK+ tdEVs (as the upper bound of the normal range that we reported previously [8]), and 10 for CK− tdEVs (assuming that each class contributes 1/3 in the total tdEV counts). However, the screening of all possible cutoff values to dichotomize patients into two risk groups and their associated HR of OS are shown in the respective plots of HRs over the CK+/− CTC and tdEV distributions (Additional file 5).

CK− CTCs (or tdEVs) perform similarly to CK+ CTCs (or tdEVs) in predicting the event of the patients as shown by the patient stratification and the resulting HR with patients with increased CK−/CK+ CTCs/tdEVs having the tendency to have worse clinical outcome compared to the patients with lower counts in their blood.

The univariable Cox regression of the continuous log-transformed CK+ and CK− CTC and tdEV counts (Supplementary Table S2, Additional file 3) showed that only CK+ tdEVs are significant predictors of OS.

### Heterogeneity of CTC/tdEV immunophenotypes and association with clinical outcome

**Fig. 3:**
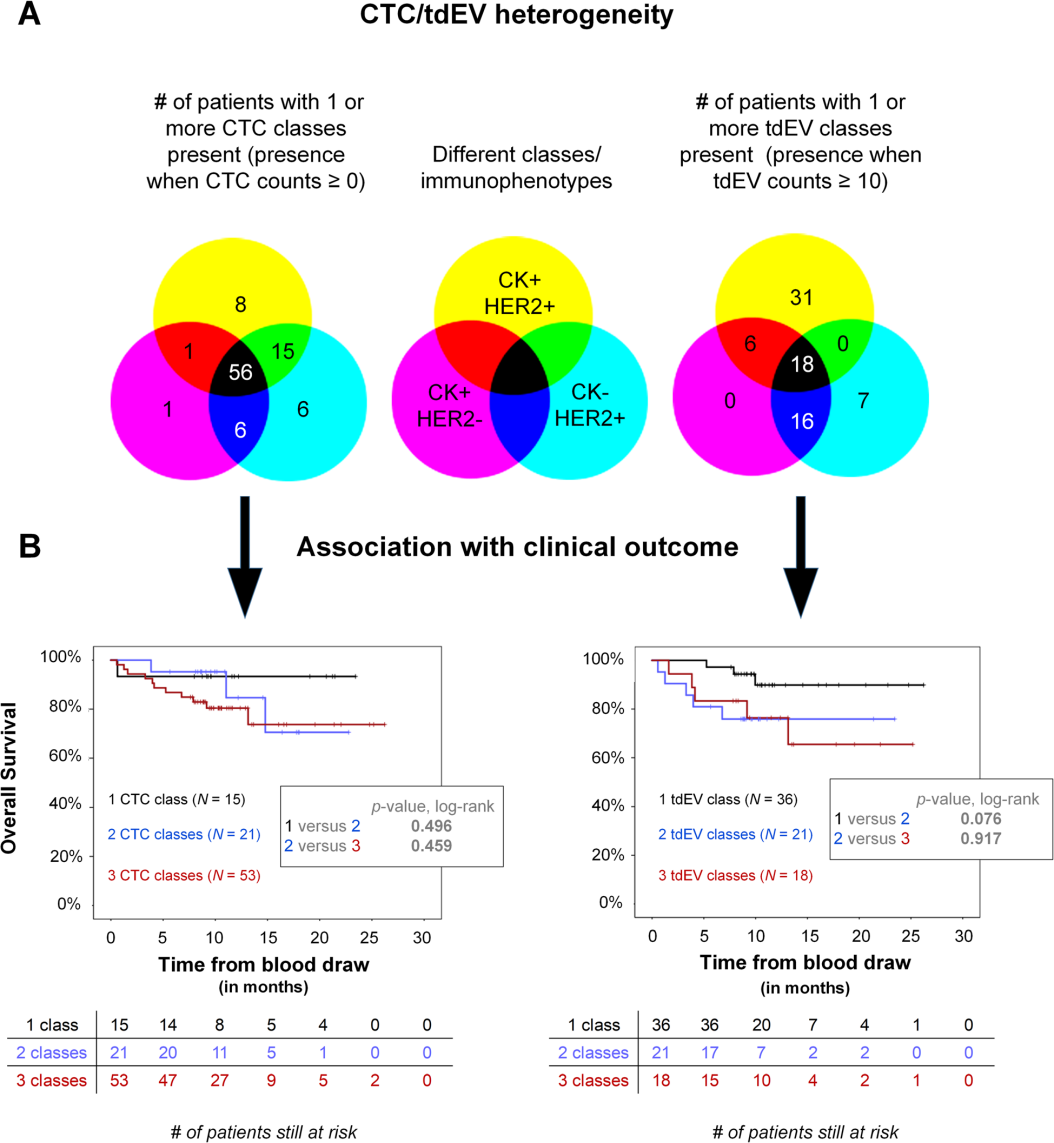
Inter- and intra-patient CTC and tdEV heterogeneity (**a**) and association with clinical outcome (**b**). The numbers of patients with the respective CTC (left) and tdEV (right) classes present in their blood are shown as numbers in the corresponding parts of the Venn diagrams (**a**). For a tdEV class to be considered present, a threshold of 10 tdEV counts was used. Patients with uniform CTC and tdEV immunophenotypes (1 class) have a better prognosis compared to patients with more heterogeneous CTC and tdEV immunophenotypes (2 or 3 classes) present (**b**)

Inter- and intra-patient heterogeneity was observed regarding the CK and HER2 phenotype of CTCs and tdEVs. The Venn diagrams in Fig. 3a show the number of patients with one or more subclasses (CK+HER2−, CK+HER2+, and CK−HER2+) of CTCs or tdEVs detected. Interestingly, the majority of patients had all three subclasses of CTCs present, whereas very few patients had solely one class of CTCs. More specifically, 56/93 (60%) of patients had CTCs of all three immunophenotypes, followed by 22/93 (24%) of patients with CTCs of 2 different immunophenotypes and only 15/93 (16%) of patients with only 1 class of CTCs present. In case of tdEVs, a threshold of 10 was considered to be the “noise,” and only when a class was present in a frequency above that number, it was considered to be present. That led to 18/98 (18%) of patients having all 3 subclasses of tdEVs present, 22/98 (22%) of them having 2 subclasses present, and 38/98 (39%) of them having one class of tdEVs present.

Patients were grouped based on the CTC/tdEV classes present in their blood, and the respective Kaplan-Meier curves of overall survival were plotted to demonstrate whereas the presence of the heterogeneous immunophenotypes is related to worse prognosis (Fig. 3b). The presence of heterogeneous immunophenotypes of CTCs/tdEVs in the blood of metastatic cancer patients (2 or 3 classes present) was associated with a worse clinical outcome compared to the patient population with a uniform CTC/tdEV immunophenotype (1 class present).

### HER2 amplification of tissue versus HER2 expression on CTCs and tdEVs

For 92 out of 98 patients, the HER2 status of the tissue was assessed by fluorescence in situ hybridization (FISH) with 39 patients (42%) having HER2+ and 53 (58%) HER2− tissue.

When we compare these 2 groups of patients in terms of their automated CTC and tdEV counts of each subclass, only CK+HER+ tdEV distribution is significantly increased (*p* < 0.05, Mann-Whitney *U* test) in HER2+ patients (Panel B, Additional file 6). The rest of the CTC and tdEV distributions (Panels A and B, Additional file 6) are not significantly different. However, the differences become more profound when comparing the 2 groups in regard to %HER2+CK+/−, %HER2+CK+, and %HER2+CK− CTCs and tdEVs estimated over the total CTC and tdEV counts, respectively. Patients with HER2+ tissue have significantly lower percentage of CK+HER2− CTCs and tdEVs and significantly higher percentage of CK+HER2+ CTCs and tdEVs compared to patients with HER2− tissues (Panels C and D, Additional file 6). No statistically significant difference could be found concerning the percentage of CK−HER2+ CTCs and tdEVs.

**Fig. 4:**
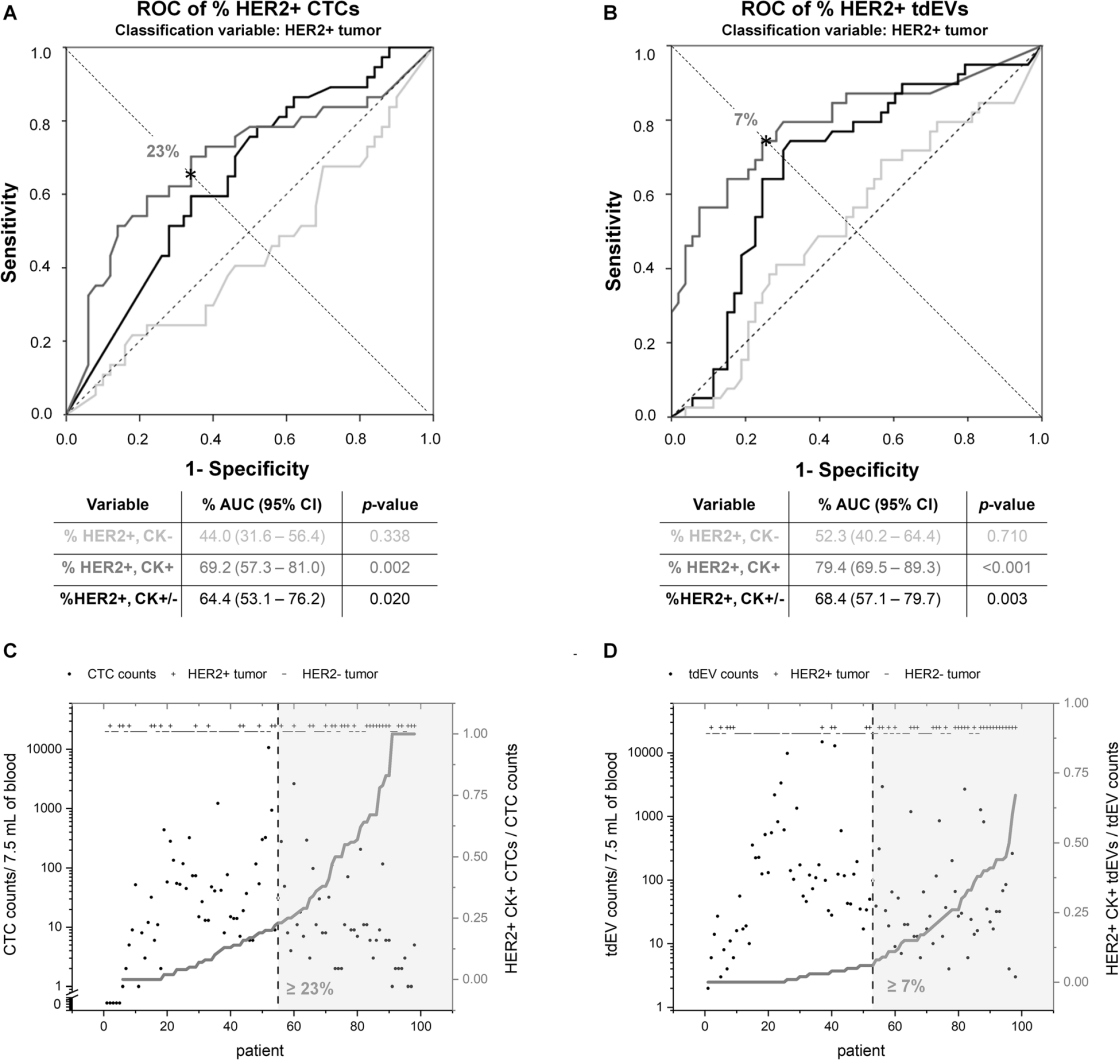
Prediction of HER2 status of tissue from CTCs and tdEVs. ROC curves of %HER2+CK− (light gray lines), HER2+CK+ (dark gray lines), and total HER2+CK+/− (black lines) CTCs (**a**) and tdEVs (**b**) treating HER2+ tissue (as assessed by FISH) as the classification variable. %HER2+CK+ populations performed the best as shown by the largest AUCs. The asterisks indicate the points, where sensitivity ≈ specificity for CTCs and tdEVs (23% HER2+CK+ CTCs leading to 65% sensitivity and 66% specificity; 7% HER2+CK+ tdEVs leading to 74% sensitivity and specificity). Scatter plot of total CTCs (**c**) and tdEVs (**d**) for each patient (*x*-axis). Samples were sorted on the percentage of HER2+CK+ CTCs or tdEVs respectively indicated by the dark gray lines. On the top of the panels and along the *x*-axis, the HER2 status of the tissue is indicated as positive (+) or negative (−), as evaluated by FISH. The vertical black dashed lines indicate the 23% HER2+ CTCs (and 7% HER2+ tdEVs) threshold, right of which the tissue of the patient could be considered as HER2+

To evaluate which HER2+ population is in a better concordance with the HER2+ primary tumor, ROC curves of the different HER2+ CTC (Fig. 4a) and tdEV (Fig. 4b) proportions were constructed treating HER2+ tissue as the classification variable. %HER2+CK+ CTCs and tdEVs performed better as indicated by the larger AUCs (0.69 for CTCs and 0.79 for tdEVs, which were not significantly different, DeLong test). The asterisks in panels a and b indicate the selected threshold of %HER2+CK+ CTCs and tdEVs, for which the test has sensitivity ≈ specificity. In case of CTCs, more than 23% HER2+CK+ CTCs (Fig. 4c) could predict a HER2+ tissue with a sensitivity of 65% and specificity of 66%. Likewise for tdEVs, more than 7% HER2+CK+ tdEVs (Fig. 4d) could predict HER2+ tissue with a sensitivity and specificity of 74%. The influence of the increasing number of CTCs and tdEVs on the accuracy of the respective test is summarized in Supplementary Table S3, Additional file 3. The accuracy of ≥ 23% HER2+CK+ CTC test improved and reached 80% when ≥ 50 CTCs were detected; however, only 27/98 (28%) patients accounted for that CTC load. In case of %HER2+CK+ tdEVs, the accuracy of the test was constantly above 70% and gradually improved from 74 up to 91% with increasing tdEV counts.

## Discussion

The HER2 is found overexpressed in around 15–30% of breast cancer cases mainly because of the HER2/neu oncogene amplification [15, 16]. Randomized clinical trials have demonstrated its predictive and prognostic value with HER2+ breast cancer patients having a remarkably improved clinical outcome when treated with anti-HER2 therapy, such as trastuzumab or lapatinib next to chemotherapy, as compared to HER2+ patients treated with solely chemotherapy [17–20]. Furthermore, HER2− breast cancer patients randomized in two treatment arms with and without anti-HER2 therapy did not show any clinical benefits further supporting the predictive value of HER2 [21]. These observations necessitate the use of an accurate test to assess the HER2 tumor status and facilitate the clinician’s treatment decision-making. The current state of the art evaluates HER2 status on solid biopsies (either tumor needle biopsy or whole tumor after resection) by immunohistochemistry or/and FISH. Several guidelines have improved the accuracy of HER2 evaluation [22]. Nevertheless, there are cases of discorcondant immunohistochemistry and FISH results or high HER2 heterogeneity of the tumor [23, 24] preventing an objective consensus on HER2 status among different operators.

Non-invasive liquid biopsies have emerged to be promising alternatives to solid biopsies providing clinically relevant tumor material in real-time. The increasing load of CK+ CTCs and tdEVs as identified by the CellSearch system is strongly associated with worsening progression-free and overall survival [1, 2, 8, 10, 25]. The CellSearch system immunomagnetically enriches CTCs targeting the epithelial cell adhesion molecule (EpCAM); consequently, all EpCAM-negative or low expressing CTCs are missed by the system. Many approaches have been introduced to overcome this limitation by enrichment of CTCs based on their physical properties such as size, density, and charge [26], but no obvious advantages have been demonstrated in clinical studies so far. Importantly, EpCAM^low^, CK+ CTCs identified after size-based separation of EpCAM-depleted blood samples did not correlate with clinical outcomes in metastatic non-small cell lung, prostate, and breast cancer [27, 28]. In the present study, we investigated whether the expression of HER2 can be assessed through tdEVs and whether CTCs and tdEVs are missed by the system due to the lack of CK expression. The whole workflow from EpCAM enrichment to CTC and tdEV scoring can be done in a fully automated manner, once a blood sample of 7.5 mL is available. Consequently, the test is not subjected to the quality of the sample, the handling, the staining methodology, and the judgment of the technician. It can be performed in a timely fashion and can facilitate the treatment monitoring of the patient.

The present analysis validates the association of CK+ tdEVs with the overall survival of metastatic breast cancer patients since it is based on an independent patient cohort than the one that we previously reported (IMMC01 study) [8]. HER2 expression could be detected on CK+ tdEVs that were co-isolated with CTCs using the CellSearch system.

The ACCEPT software allows the visualization and automated enumeration of CK− objects, which are not presented to the operator by the FDA-cleared CellSearch image analysis algorithm (CellTracks Analyzer II). Interestingly, the inclusion of the HER2-FITC antibody allowed the detection of CD45−, CK−, and HER2+ CTCs and tdEVs in the EpCAM-enriched blood samples of metastatic breast cancer patients increasing the percentage of patient samples with detectable CTCs from 89 to 95%. CK+ tdEVs were already present in 99% of patient samples, which increased to 100% after the inclusion of anti-HER2.

Although we have not genetically proven that these CK− CTCs and tdEVs are indeed cancerous, their similar correlation with OS (Fig. 2) tends to confirm that hypothesis. The aneuploidy of these CK− cells should be addressed by FISH in a prospective clinical study, since the cartridges, whose image datasets were used in the present analysis, are no longer available. Fehm et al. [29] first demonstrated the malignancy of EpCAM-enriched CK+, CD45−, and DAPI+ cells (CellSearch CTC definition) by FISH from the blood of patients with carcinomas. A protocol to perform FISH directly in the CellSearch cartridges was developed later by Swennenhuis et al. [30], who evaluated the technique in blood samples of castration-resistant prostate cancer patients.

The observation of CK−HER2+ CTCs and tdEVs raises questions about other CK−HER2− CTC and tdEV populations present in the EpCAM-enriched samples. The findings of Crespo et al. that aneuploid CD45−, CK−, and AR+ CTCs in EpCAM-enriched blood samples of castration-resistant prostate cancer patients are associated with worse OS further support our hypothesis [31]. The detection of CK− aneuploid cells in blood samples of ovarian, breast, and colorectal cancer patients has been also described by Pecot et al. [32]. All aforementioned studies further encourage the inclusion of additional antibodies to detect nucleated events of unknown cell lineage in the EpCAM-enriched samples and decrease false-negative rates of CTCs.

Importantly, all three immunophenotypes of CTCs (HER2+CK−, HER2−CK+, and HER2+CK+) were present in the majority of EpCAM-enriched patient samples, and the presence of heterogeneous CTC and tdEV immunophenotypes was associated with worse clinical outcome of the patients (Fig. 3).

Our next question was whereas a minimum threshold of a HER2+ population could predict the HER2 status of the tissue as a real-time liquid biopsy. The %HER2+CK+ tdEVs performed the best achieving higher sensitivity and specificity than CTCs (Fig. 4), most likely because of the higher frequencies of tdEVs better reflecting the tumor heterogeneity. Based on that test, a patient can be affirmed to have a HER2+ tumor with at least 74% accuracy if ≥ 7% of total EpCAM+ tdEVs are CK+ and HER2+. However, it is important to mention that the determination of the HER2 amplification status of the primary tissue was done at the time of diagnosis with most of the patients being as stages I–III. The CTC evaluation reported in the original study (and the present analysis) occurred after the progression of the patients to stage IV (one of the inclusion criteria of the patients enrolled in the study), which can be years after the initial diagnosis and the tumor resection. It is well known that the phenotype and genotype of the primary tumor can substantially differ from the respective genotypic and phenotypic features of the metastatic lesions as the disease progresses [33–36] with CTCs resembling better the mutational status of metastatic lesions than of primary tumor [37]. Therefore, the HER2 expression on the primary tumor can differ substantially from the status of the metastatic lesions as well the CTCs and tdEVs isolated having an impact on the concordance found. Whether the HER2 assessment via a liquid biopsy (CTCs or/and tdEVs) can better predict response to anti-HER2-targeted therapies compared to the current assessment via a solid biopsy remains to be addressed.

Molecular characterization of the EpCAM+ tdEVs of patients undergoing HER2-targeted therapies at follow-up time points can contribute to better comprehend the underlying mechanism of tumor resistance to anti-HER2 treatment [38], which is observed in more than 70% of HER+ breast cancer patients within a year from the initiation of the treatment [39]. Ciravolo et al. have already suggested a mechanism of anti-HER2 resistance by the increased binding efficiency of HER2+ exosomes to trastuzumab in progressive HER2+ breast cancers as compared to earlier stages of breast cancer [40]. Another mechanism has been suggested by Al-Nedawi et al. with tdEVs transferring the oncogenic form of epidermal growth factor receptor EGFRvIII to cells without that immunophenotype [41].

## Conclusions

The inclusion of anti-HER2 in the CellSearch assay allowed the detection of EpCAM+CK− CTCs and tdEVs with similar prognostic power as EpCAM+CK+ CTCs and tdEVs in metastatic breast cancer emerging the importance of including more detection antibodies next to CK for the identification of CK− CTCs and tdEVs. Importantly, the presence of heterogeneous CTC and tdEVs (2 or 3 different immunophenotypes) in the blood of cancer patients was associated with worse overall survival compared to patients with uniform CTCs and tdEVs (1 immunophenotype). These findings should be validated in a prospective study, where also the malignancy of these populations should be addressed by FISH. Our results enrich the already available data of real-time liquid biopsy and encourage the screening of tdEVs for treatment targets in clinics, since they seem to better reflect the HER2 phenotype of the primary tumor than CTCs opening the path towards a more rational and objective choice of the patients who will or will not be subjected to HER2 targeting therapies.

## Supplementary information

**Additional file 1: Figure S1.** Comparison between the patient subpopulation used in the present analysis and the complete patient cohort of the CLCC-IC-2006-04 study. Probability distribution for Overall Survival of complete patient cohort (in black) and patient subpopulation used (in pink) as shown by the respective Kaplan Meier plots (Panel A). Box plot with overlapping data of manual CTC counts of complete patient cohort (in black) and patient subpopulation used (in pink).

**Additional file 2: Figure S2.** ACCEPT display of different subclasses of CTCs (left) and tdEVs (right) isolated by the CellSearch system from metastatic breast cancer patients. Examples of CK+HER2- (Panel A), CK-HER2+ (Panel B) and CK+HER2+ (Panel C) CTCs and tdEVs. The red contours around the objects in the respective channel indicate the detected and segmented signal by the ACCEPT image analysis algorithm. All objects were isolated from 7.5 mL of blood using the CellSearch system. Scale bars indicate 6.4 μm.

**Additional file 3: Supplementary Table S1.** Linear ACCEPT gates used for the automated enumeration of CTCs and tdEVs. **Supplementary Table S2.** Univariable Cox regression analyses of CK+ and CK- CTCs and tdEVs after log transformation. **Supplementary Table S3.** Sensitivity, specificity and accuracy of ≥ 23% CTCs and ≥ 7% tdEVs, double positive for CK and HER2, as tests to predict the HER2 status of the tissue. The accuracy increases with the total CTCs and tdEVs detected (≥ 1, 5, 10, 20, 50, 100) at the cost of number of eligible patients to be assessed.

**Additional file 4: Figure S3.** Correlation of manual with automated CK+ CTC counts and association with clinical outcome of patients. Scatter plot of CK+ manual CTCs (mCTCs) versus CK+ automated CTCs (aCTCs) showing strong correlation (Panel A). KM plots of OS (Panel B) for patients with < and ≥ 5 CTCs. The dichotomization of patients was done based on either manual (black and grey lines) or automated (red and green) CTC counts showing equivalent association to OS.

**Additional file 5: Figure S4.** Overview plots of HRs (with 95% CI) for all possible cut-off values for CK+ CTCs (Panel A), CK+ tdEVs (Panel B), CK- CTCs (Panel C) and CK- tdEVs (Panel D). The rug plots at the bottom of Panels A-D correspond to the value distributions of CK+ CTCs, CK+ tdEVs, CK- CTCs and CK- tdEVs respectively. For CK+ CTCs, a larger percentage of cut-off values (31%, Panel A) could significantly dichotomize patients with a higher and lower risk as compared to CK- CTCs (13%, Panel C). The opposite was observed for tdEVs with a larger percentage of cut-off values for CK- tdEVs (30%, Panel D) leading to a significant dichotomization of patients as compared to CK+ tdEVs (14%, Panel B).

**Additional file 6: Figure S5.** Comparison of patients with HER2+ and HER2- tissues in regards to their relative and absolute frequencies of CTCs and tdEVs of different phenotypes. Box plots with data overlap depicting the automated CTC counts (Panel A), automated tdEV counts (Panel B), % of CTCs (Panel C) and % of tdEVs (Panel D) of the 3 different immunophenotypes (indicated in the x-axis) in metastatic breast cancer patients split based on the HER2 status of their tissue. Each dot corresponds to the counts of one patient (green dots: patients with HER2+ tissue (*N*=39), red dots: patients with HER2- tissue (*N*= 53), there was no available HER2 status for *N*=6 patients). Lower and upper bounds of box plots correspond to the 1st (Q1) and 3rd (Q3) quartile of data, horizontal black lines indicate median vaues. Whiskers indicate 1.5*(Q3-Q1). * indicates significant (*p* < 0.05) and ** highly significant (*p* < 0.001) statistical difference (Mann-Whitney U test).

## Data Availability

The datasets used and analyzed during the current study are available from the corresponding author on reasonable request.

## Abbreviations

ACCEPT: Automated CTC Classification, Enumeration and PhenoTyping
AR: Androgen receptor
AUC: Area under the curve
CIs: Confidence intervals
CK: Cytokeratin
CTCs: Circulating tumor cells
EGFRvIII: Epidermal growth factor receptor variant III
EpCAM: Epithelial cell adhesion molecule
FDA: Food and Drug Administration
FISH: Fluorescence in situ hybridization
FITC: Fluorescein isothiocyanate
HER2: Human epidermal growth factor receptor 2
HRs: Hazard ratios
KM: Kaplan-Meier
OS: Overall survival
ROC: Receiver operating characteristic
tdEVs: Tumor-derived extracellular vesicles

## References

[1] Miller MC, Doyle GV, Terstappen LW (2010). Significance of circulating tumor cells detected by the CellSearch system in patients with metastatic breast colorectal and prostate cancer. J Oncol.

[2] Lindsay CR, Blackhall FH, Carmel A, Fernandez-Gutierrez F, Gazzaniga P, Groen HJM, Hiltermann TJN, Krebs MG, Loges S, Lopez-Lopez R (2019). EPAC-lung: pooled analysis of circulating tumour cells in advanced non-small cell lung cancer. Eur J Cancer.

[3] Ligthart ST, Bidard FC, Decraene C, Bachelot T, Delaloge S, Brain E, Campone M, Viens P, Pierga JY, Terstappen LW (2013). Unbiased quantitative assessment of Her-2 expression of circulating tumor cells in patients with metastatic and non-metastatic breast cancer. Ann Oncol.

[4] Onstenk W, Gratama JW, Foekens JA, Sleijfer S (2013). Towards a personalized breast cancer treatment approach guided by circulating tumor cell (CTC) characteristics. Cancer Treat Rev.

[5] Zeune L, van Dalum G, Decraene C, Proudhon C, Fehm T, Neubauer H, Rack B, Alunni-Fabbroni M, Terstappen LWMM, van Gils SA (2017). Quantifying HER-2 expression on circulating tumor cells by ACCEPT. PLoS One.

[6] Zeune LL, de Wit S, Berghuis AMS, Ijzerman MJ, Terstappen LWMM, Brune C (2018). How to agree on a CTC: evaluating the consensus in circulating tumor cell scoring. Cytometry A.

[7] Nanou A, Coumans FAW, van Dalum G, Zeune LL, Dolling D, Onstenk W, Crespo M, Fontes MS, Rescigno P, Fowler G, et al. Circulating tumor cells, tumor-derived extracellular vesicles and plasma cytokeratins in castration-resistant prostate cancer patients. Oncotarget. 2018;9(27):19283–93. 10.18632/oncotarget.25019 PMC5922396 29721202

[8] Nanou A, Miller MC, Zeune LL, de Wit S, Punt CJA, Groen HJM, Hayes DF, de Bono JS, Terstappen L. Tumour-derived extracellular vesicles in blood of metastatic cancer patients associate with overall survival. Br J Cancer. 2020;122(6):801–11. 10.1038/s41416-019-0726-9 PMC7078322 31937922

[9] Pierga JY, Hajage D, Bachelot T, Delaloge S, Brain E, Campone M, Dieras V, Rolland E, Mignot L, Mathiot C (2012). High independent prognostic and predictive value of circulating tumor cells compared with serum tumor markers in a large prospective trial in first-line chemotherapy for metastatic breast cancer patients. Ann Oncol.

[10] Ligthart ST, Coumans FAW, Attard G, Cassidy AM, de Bono JS, Terstappen LWMM (2011). Unbiased and automated identification of a circulating tumour cell definition that associates with overall survival. PLoS One.

[11] Hayes DF, Walker TM, Singh B, Vitetta ES, Uhr JW, Gross S, Rao C, Doyle GV, Terstappen LW (2002). Monitoring expression of HER-2 on circulating epithelial cells in patients with advanced breast cancer. Int J Oncol.

[12] Zeune L, van Dalum G, Terstappen LWMM, van Gils SA, Brune C (2017). Multiscale segmentation via Bregman distances and nonlinear spectral analysis. Siam J Imaging Sci.

[13] Budczies J, Klauschen F, Sinn BV, Gyorffy B, Schmitt WD, Darb-Esfahani S, Denkert C (2012). Cutoff finder: a comprehensive and straightforward Web application enabling rapid biomarker cutoff optimization. PLoS One.

[14] DeLong ER, DeLong DM, Clarke-Pearson DL (1988). Comparing the areas under two or more correlated receiver operating characteristic curves: a nonparametric approach. Biometrics.

[15] Slamon DJ, Clark GM, Wong SG, Levin WJ, Ullrich A, McGuire WL (1987). Human breast cancer: correlation of relapse and survival with amplification of the HER-2/neu oncogene. Science (New York, N Y ).

[16] Gullick WJ, Love SB, Wright C, Barnes DM, Gusterson B, Harris AL, Altman DG (1991). c-erbB-2 protein overexpression in breast cancer is a risk factor in patients with involved and uninvolved lymph nodes. Br J Cancer.

[17] Geyer CE, Forster J, Lindquist D, Chan S, Romieu CG, Pienkowski T, Jagiello-Gruszfeld A, Crown J, Chan A, Kaufman B (2006). Lapatinib plus capecitabine for HER2-positive advanced breast cancer. N Engl J Med.

[18] Gianni L, Eiermann W, Semiglazov V, Manikhas A, Lluch A, Tjulandin S, Zambetti M, Vazquez F, Byakhow M, Lichinitser M (2010). Neoadjuvant chemotherapy with trastuzumab followed by adjuvant trastuzumab versus neoadjuvant chemotherapy alone, in patients with HER2-positive locally advanced breast cancer (the NOAH trial): a randomised controlled superiority trial with a parallel HER2-negative cohort. Lancet (London, England).

[19] Piccart-Gebhart MJ, Procter M, Leyland-Jones B, Goldhirsch A, Untch M, Smith I, Gianni L, Baselga J, Bell R, Jackisch C (2005). Trastuzumab after adjuvant chemotherapy in HER2-positive breast cancer. N Engl J Med.

[20] Baselga J, Bradbury I, Eidtmann H, Di Cosimo S, de Azambuja E, Aura C, Gomez H, Dinh P, Fauria K, Van Dooren V (2012). Lapatinib with trastuzumab for HER2-positive early breast cancer (NeoALTTO): a randomised, open-label, multicentre, phase 3 trial. Lancet (London, England).

[21] Di Leo A, Gomez HL, Aziz Z, Zvirbule Z, Bines J, Arbushites MC, Guerrera SF, Koehler M, Oliva C, Stein SH (2008). Phase III, double-blind, randomized study comparing lapatinib plus paclitaxel with placebo plus paclitaxel as first-line treatment for metastatic breast cancer. J Clin Oncol.

[22] Wolff AC, Hammond MEH, Allison KH, Harvey BE, Mangu PB, Bartlett JMS, Bilous M, Ellis IO, Fitzgibbons P, Hanna W (2018). Human epidermal growth factor receptor 2 testing in breast cancer: American Society of Clinical Oncology/College of American Pathologists Clinical Practice Guideline Focused Update. J Clin Oncol.

[23] Buckley NE, Forde C, McArt DG, Boyle DP, Mullan PB, James JA, Maxwell P, McQuaid S, Salto-Tellez M (2016). Quantification of HER2 heterogeneity in breast cancer-implications for identification of sub-dominant clones for personalised treatment. Sci Rep.

[24] Nitta H, Kelly BD, Allred C, Jewell S, Banks P, Dennis E, Grogan TM (2016). The assessment of HER2 status in breast cancer: the past, the present, and the future. Pathol Int.

[25] de Wit S, Rossi E, Weber S, Tamminga M, Manicone M, Swennenhuis JF, Groothuis-Oudshoorn CGM, Vidotto R, Facchinetti A, Zeune LL (2019). Single tube liquid biopsy for advanced non-small cell lung cancer. Int J Cancer.

[26] Harouaka R, Kang Z, Zheng SY, Cao L (2014). Circulating tumor cells: advances in isolation and analysis, and challenges for clinical applications. Pharmacol Ther.

[27] de Wit S, Manicone M, Rossi E, Lampignano R, Yang L, Zill B, Rengel-Puertas A, Ouhlen M, Crespo M, Berghuis AMS (2018). EpCAM^high^ and EpCAM^low^ circulating tumor cells in metastatic prostate and breast cancer patients. Oncotarget.

[28] De Wit S, van Dalum G, Lenferink ATM, Tibbe AGJ, Hiltermann TJN, Groen HJM, van Rijn CJM, Terstappen LWMM. The detection of EpCAM^+^ and EpCAM^−^ circulating tumor cells. Sci Rep-Uk. 2015;5:12270. 10.1038/srep12270 PMC4505332 26184843

[29] Fehm T, Sagalowsky A, Clifford E, Beitsch P, Saboorian H, Euhus D, Meng S, Morrison L, Tucker T, Lane N, et al. Cytogenetic evidence that circulating epithelial cells in patients with carcinoma are malignant. Clin Cancer Res. 2002;8(7):2073–84. 12114406

[30] Swennenhuis JF, Tibbe AG, Levink R, Sipkema RC, Terstappen LW (2009). Characterization of circulating tumor cells by fluorescence in situ hybridization. Cytometry A.

[31] Crespo M, van Dalum G, Ferraldeschi R, Zafeiriou Z, Sideris S, Lorente D, Bianchini D, Rodrigues DN, Riisnaes R, Miranda S (2015). Androgen receptor expression in circulating tumour cells from castration-resistant prostate cancer patients treated with novel endocrine agents. Br J Cancer.

[32] Pecot CV, Bischoff FZ, Mayer JA, Wong KL, Pham T, Bottsford-Miller J, Stone RL, Lin YG, Jaladurgam P, Roh JW (2011). A novel platform for detection of CK+ and CK- CTCs. Cancer Discov.

[33] Pestrin M, Bessi S, Galardi F, Truglia M, Biggeri A, Biagioni C, Cappadona S, Biganzoli L, Giannini A, Di Leo A (2009). Correlation of HER2 status between primary tumors and corresponding circulating tumor cells in advanced breast cancer patients. Breast Cancer Res Treat.

[34] Onstenk W, Sieuwerts AM, Weekhout M, Mostert B, Reijm EA, van Deurzen CH, Bolt-de Vries JB, Peeters DJ, Hamberg P, Seynaeve C (2015). Gene expression profiles of circulating tumor cells versus primary tumors in metastatic breast cancer. Cancer Lett.

[35] Meng S, Tripathy D, Shete S, Ashfaq R, Haley B, Perkins S, Beitsch P, Khan A, Euhus D, Osborne C (2004). HER-2 gene amplification can be acquired as breast cancer progresses. Proc Natl Acad Sci.

[36] Fehm T, Becker S, Duerr-Stoerzer S, Sotlar K, Mueller V, Wallwiener D, Lane N, Solomayer E, Uhr J (2007). Determination of HER2 status using both serum HER2 levels and circulating tumor cells in patients with recurrent breast cancer whose primary tumor was HER2 negative or of unknown HER2 status. Breast Cancer Res.

[37] Onstenk W, Sieuwerts AM, Mostert B, Lalmahomed Z, Bolt-de Vries JB, van Galen A, Smid M, Kraan J, Van M, de Weerd V (2016). Molecular characteristics of circulating tumor cells resemble the liver metastasis more closely than the primary tumor in metastatic colorectal cancer. Oncotarget.

[38] Pohlmann PR, Mayer IA, Mernaugh R (2009). Resistance to trastuzumab in breast cancer. Clin Cancer Res.

[39] Vogel CL, Cobleigh MA, Tripathy D, Gutheil JC, Harris LN, Fehrenbacher L, Slamon DJ, Murphy M, Novotny WF, Burchmore M (2002). Efficacy and safety of trastuzumab as a single agent in first-line treatment of HER2-overexpressing metastatic breast cancer. J Clin Oncol.

[40] Ciravolo V, Huber V, Ghedini GC, Venturelli E, Bianchi F, Campiglio M, Morelli D, Villa A, Della Mina P, Menard S (2012). Potential role of HER2-overexpressing exosomes in countering trastuzumab-based therapy. J Cell Physiol.

[41] Al-Nedawi K, Meehan B, Micallef J, Lhotak V, May L, Guha A, Rak J (2008). Intercellular transfer of the oncogenic receptor EGFRvIII by microvesicles derived from tumour cells. Nat Cell Biol.

