# Supplementary figures and images for: HER2 expression on tumor-derived extracellular vesicles and circulating tumor cells in metastatic breast cancer
Source: Breast Cancer Res. 2020 Aug 12;22:86. doi: 10.1186/s13058-020-01323-5 (PMC7424685; doi:10.1186/s13058-020-01323-5)

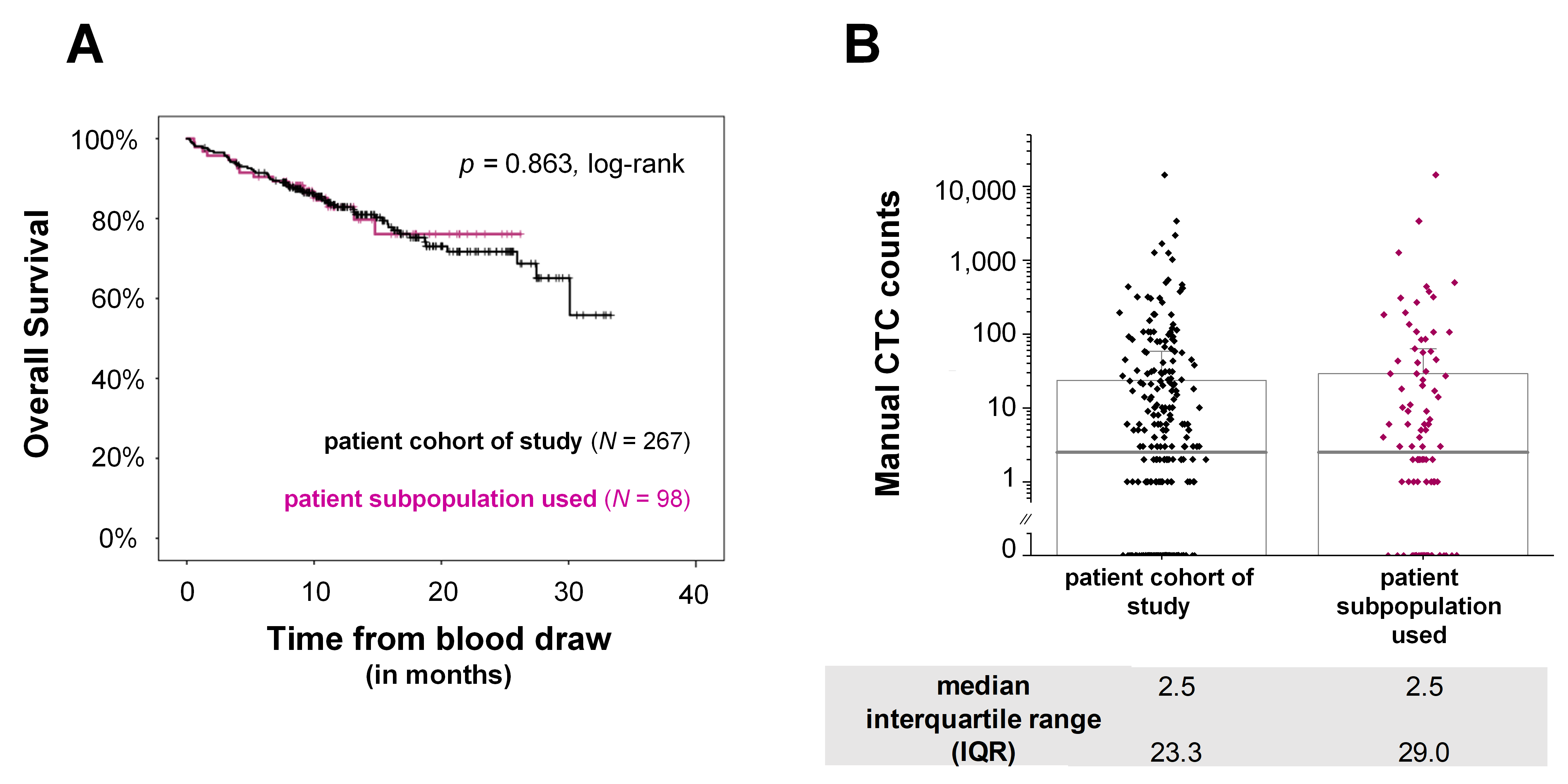

Supplement: Supplementary file 1 — Additional file 1: Figure S1. Comparison between the patient subpopulation used in the present analysis and the complete patient cohort of the CLCC-IC-2006-04 study. Probability distribution for Overall Survival of complete patient cohort (in black) and patient subpopulation used (in pink) as shown by the respective Kaplan Meier plots (Panel A). Box plot with overlapping data of manual CTC counts of complete patient cohort (in black) and patient subpopulation used (in pink). [file 13058_2020_1323_MOESM1_ESM.tiff]

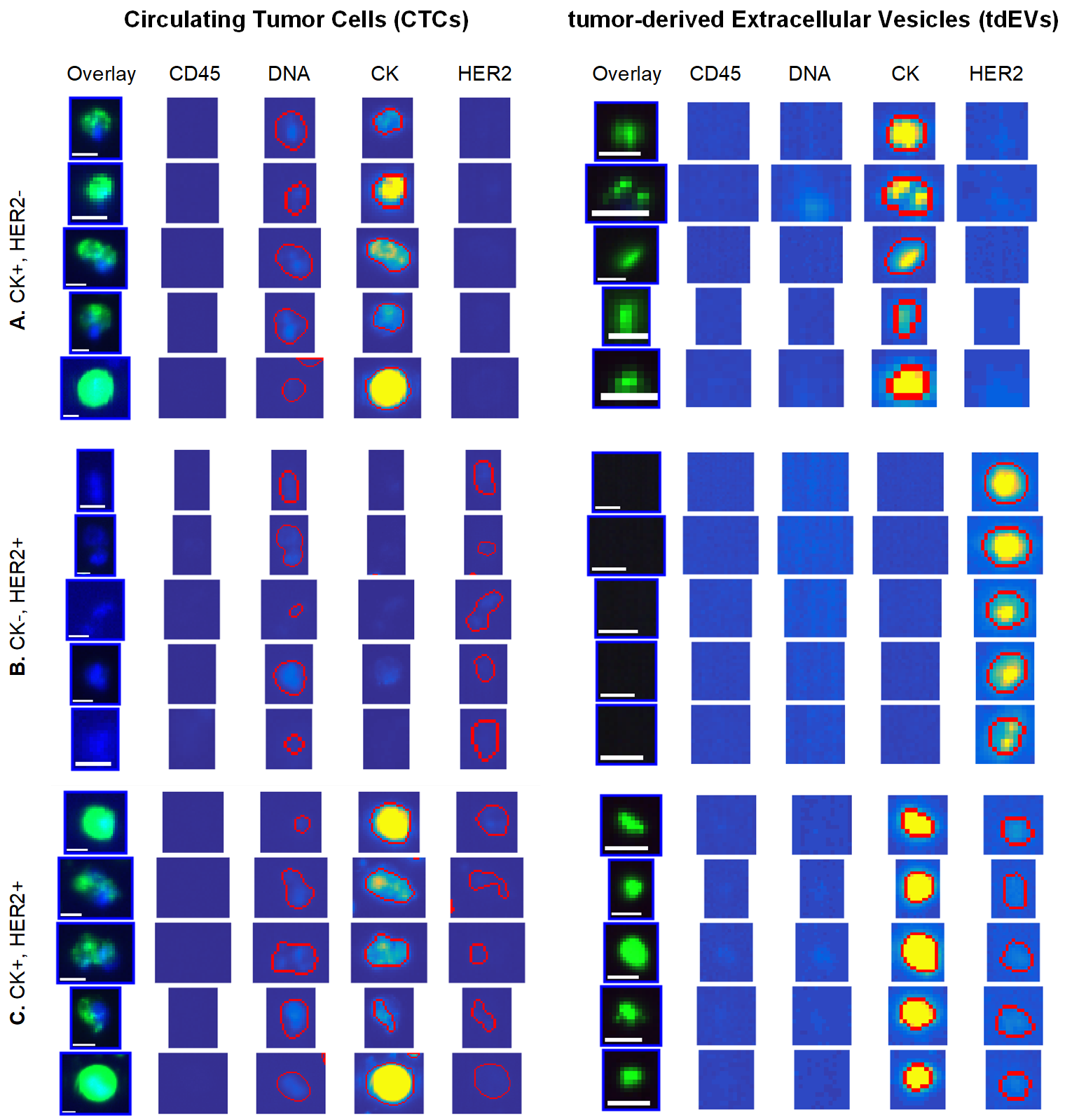

Supplement: Supplementary file 2 — Additional file 2: Figure S2. ACCEPT display of different subclasses of CTCs (left) and tdEVs (right) isolated by the CellSearch system from metastatic breast cancer patients. Examples of CK+HER2- (Panel A), CK-HER2+ (Panel B) and CK+HER2+ (Panel C) CTCs and tdEVs. The red contours around the objects in the respective channel indicate the detected and segmented signal by the ACCEPT image analysis algorithm. All objects were isolated from 7.5 mL of blood using the CellSearch system. Scale bars indicate 6.4 μm. [file 13058_2020_1323_MOESM2_ESM.tif]

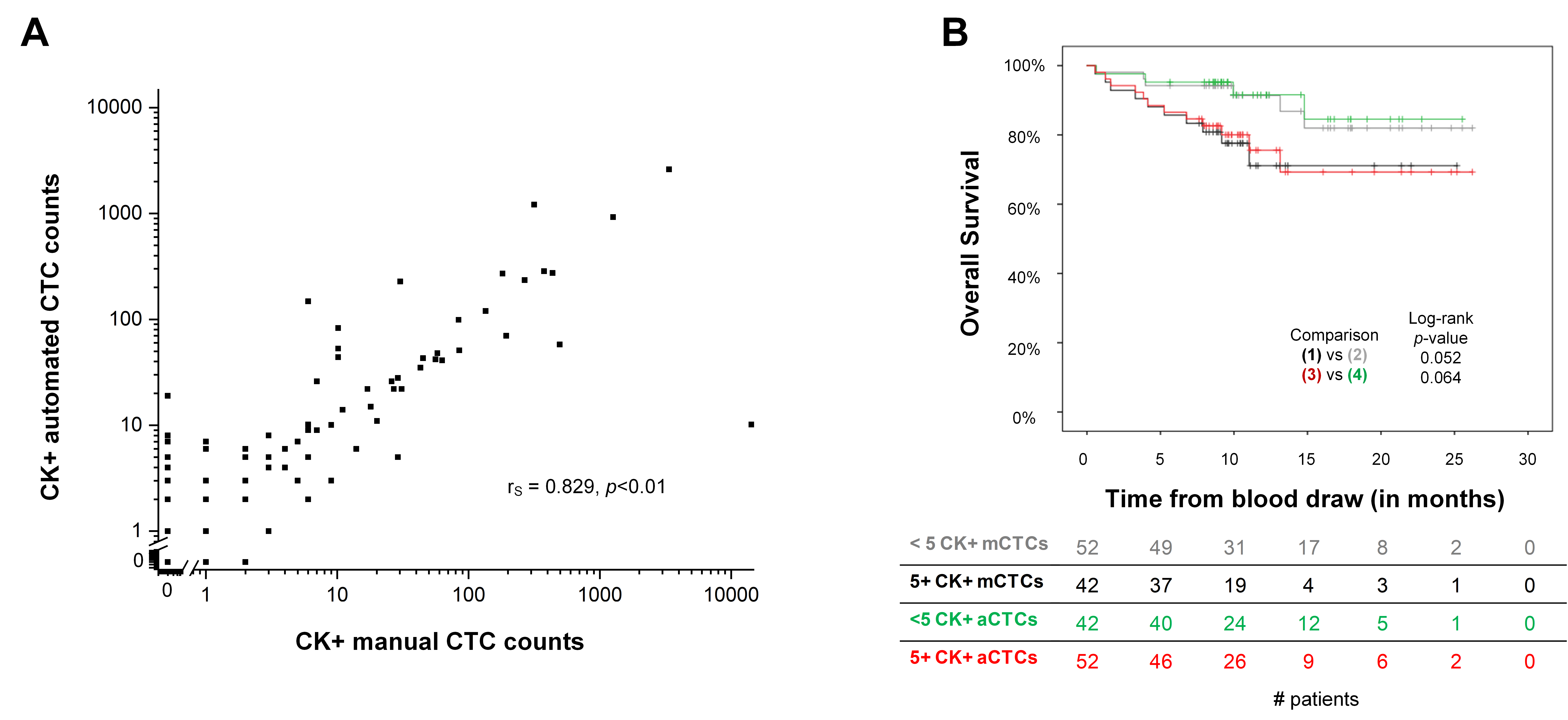

Supplement: Supplementary file 4 — Additional file 4: Figure S3. Correlation of manual with automated CK+ CTC counts and association with clinical outcome of patients. Scatter plot of CK+ manual CTCs (mCTCs) versus CK+ automated CTCs (aCTCs) showing strong correlation (Panel A). KM plots of OS (Panel B) for patients with < and ≥ 5 CTCs. The dichotomization of patients was done based on either manual (black and grey lines) or automated (red and green) CTC counts showing equivalent association to OS. [file 13058_2020_1323_MOESM4_ESM.tif]

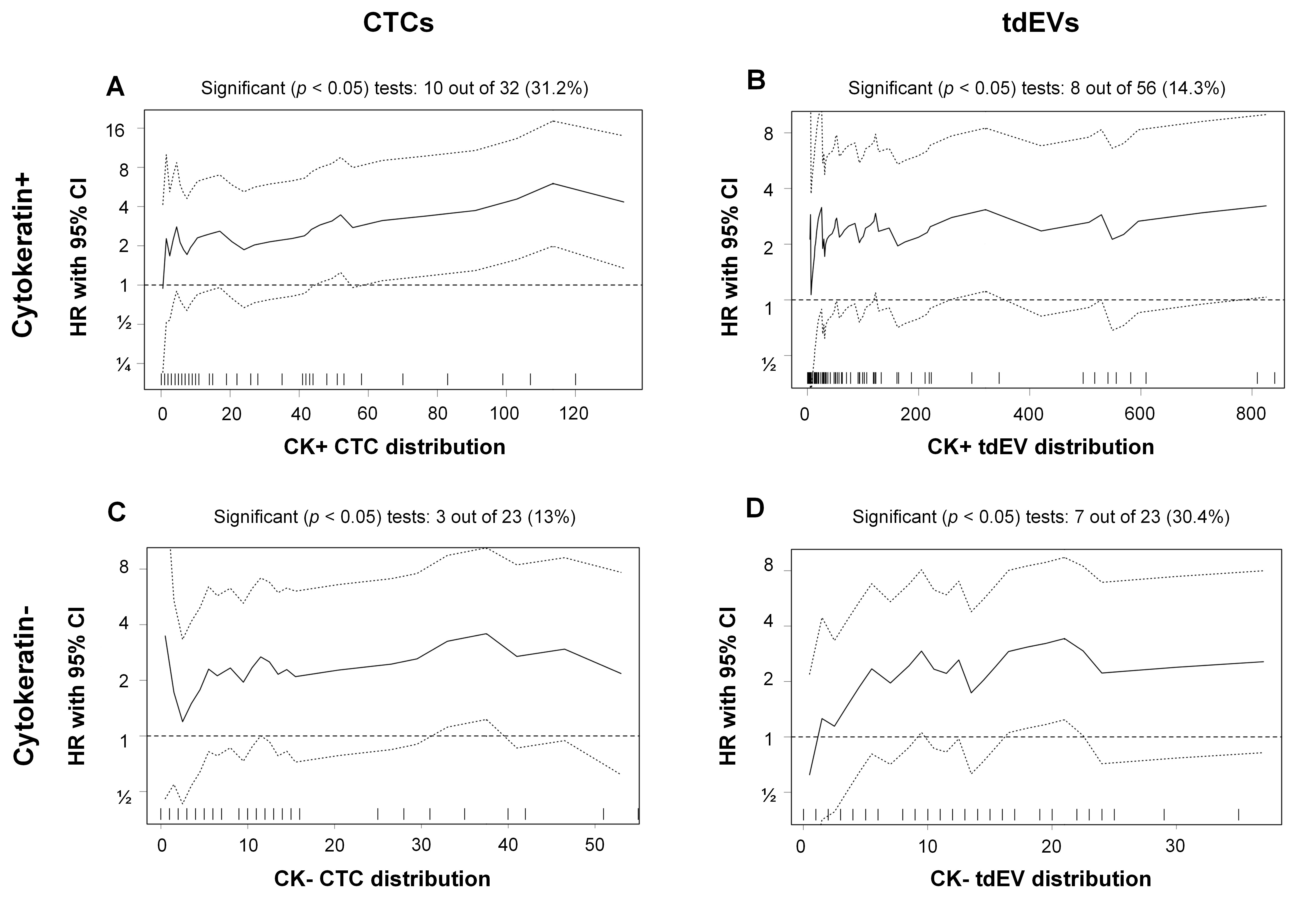

Supplement: Supplementary file 5 — Additional file 5: Figure S4. Overview plots of HRs (with 95% CI) for all possible cut-off values for CK+ CTCs (Panel A), CK+ tdEVs (Panel B), CK- CTCs (Panel C) and CK- tdEVs (Panel D). The rug plots at the bottom of Panels A-D correspond to the value distributions of CK+ CTCs, CK+ tdEVs, CK- CTCs and CK- tdEVs respectively. For CK+ CTCs, a larger percentage of cut-off values (31%, Panel A) could significantly dichotomize patients with a higher and lower risk as compared to CK- CTCs (13%, Panel C). The opposite was observed for tdEVs with a larger percentage of cut-off values for CK- tdEVs (30%, Panel D) leading to a significant dichotomization of patients as compared to CK+ tdEVs (14%, Panel B). [file 13058_2020_1323_MOESM5_ESM.tiff]

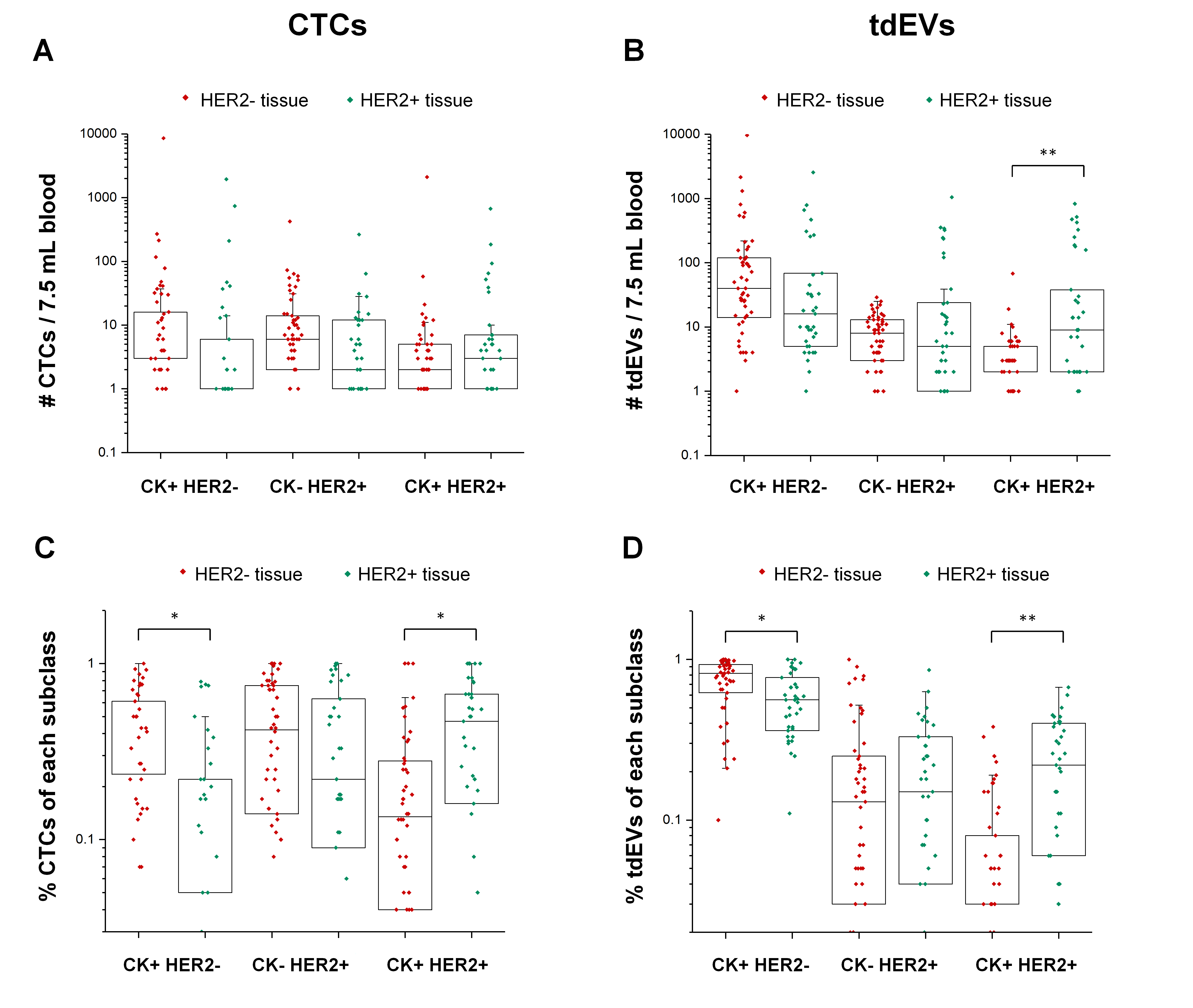

Supplement: Supplementary file 6 — Additional file 6: Figure S5. Comparison of patients with HER2+ and HER2- tissues in regards to their relative and absolute frequencies of CTCs and tdEVs of different phenotypes. Box plots with data overlap depicting the automated CTC counts (Panel A), automated tdEV counts (Panel B), % of CTCs (Panel C) and % of tdEVs (Panel D) of the 3 different immunophenotypes (indicated in the x-axis) in metastatic breast cancer patients split based on the HER2 status of their tissue. Each dot corresponds to the counts of one patient (green dots: patients with HER2+ tissue (N=39), red dots: patients with HER2- tissue (N= 53), there was no available HER2 status for N=6 patients). Lower and upper bounds of box plots correspond to the 1st (Q1) and 3rd (Q3) quartile of data, horizontal black lines indicate median vaues. Whiskers indicate 1.5*(Q3-Q1). * indicates significant (p < 0.05) and ** highly significant (p < 0.001) statistical difference (Mann-Whitney U test). [file 13058_2020_1323_MOESM6_ESM.tif]
