# Supplementary material for: HER2 expression on tumor-derived extracellular vesicles and circulating tumor cells in metastatic breast cancer
Source: Breast Cancer Res. 2020 Aug 12;22:86. doi: 10.1186/s13058-020-01323-5 (PMC7424685; doi:10.1186/s13058-020-01323-5)
Supplement: Supplementary file 3 — Additional file 3: Supplementary Table S1. Linear ACCEPT gates used for the automated enumeration of CTCs and tdEVs. Supplementary Table S2. Univariable Cox regression analyses of CK+ and CK- CTCs and tdEVs after log transformation. Supplementary Table S3. Sensitivity, specificity and accuracy of ≥ 23% CTCs and ≥ 7% tdEVs, double positive for CK and HER2, as tests to predict the HER2 status of the tissue. The accuracy increases with the total CTCs and tdEVs detected (≥ 1, 5, 10, 20, 50, 100) at the cost of number of eligible patients to be assessed. [file 13058_2020_1323_MOESM3_ESM.docx]

**Supplementary** **Table S1:** Linear ACCEPT gates used for the automated enumeration of CTCs and tdEVs.


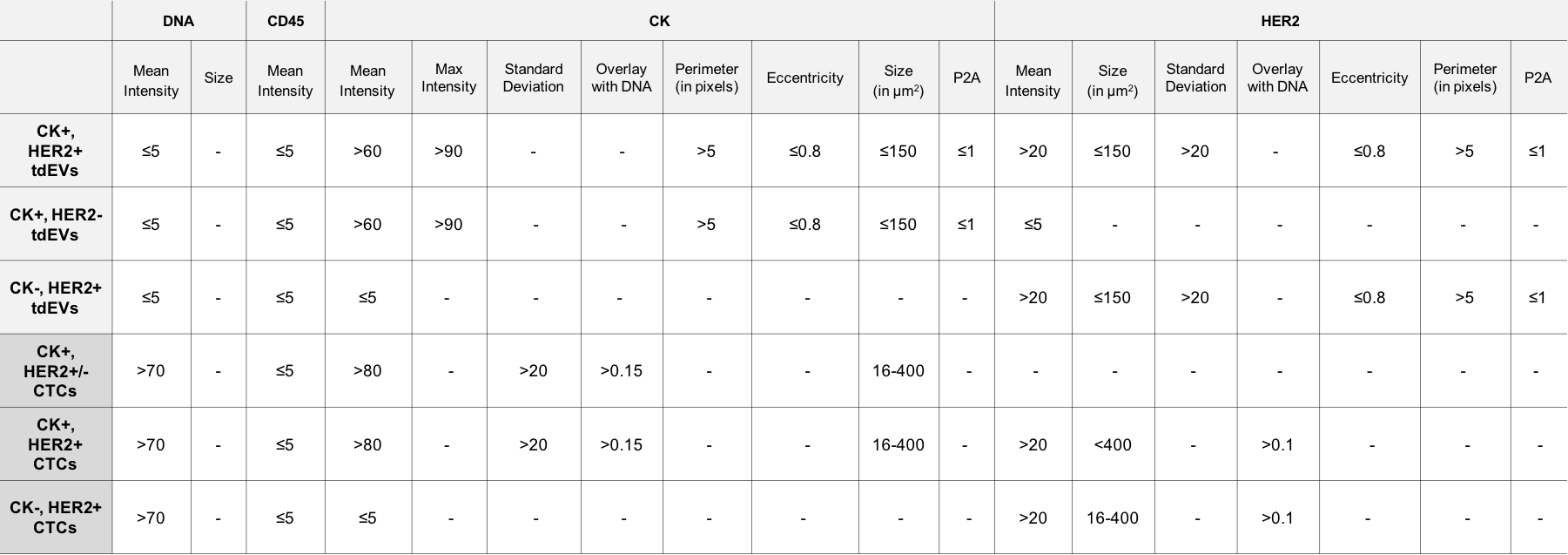


**Supplementary** **Table S2:** Univariable Cox regression analyses of CK+ and CK- CTCs and tdEVs after log transformation.

|  | ***Overall survival*** | |
| --- | --- | --- |
|  | **HR (95% CI)** | ***p*-value** |
| **log (0.1 + CK^-^ CTCs)** | 1.70 (0.94 – 3.08) | 0.081 |
| **log (0.1 + CK^+^ CTCs)** | 1.41 (0.89 – 2.24) | 0.140 |
| **log (0.1 + CK^-^ tdEVs)** | 1.32 (0.75 – 2.32) | 0.345 |
| **log (0.1 + CK^+^ tdEVs)** | 1.69 (1.03 – 2.79) | 0.039 |

**Supplementary** **Table S3: Sensitivity, specificity and accuracy of ≥ 23% CTCs and ≥ 7% tdEVs, double positive for CK and HER2, as tests to predict the HER2 status of the tissue.** The accuracy increases with the total CTCs and tdEVs detected (≥ 1, 5, 10, 20, 50, 100) at the cost of number of eligible patients to be assessed.

| **HER2+CK+ CTCs / total CTCs** | **Total CTCs** | **Patients**  ***N* (%)** | **sensitivity** | **specificity** | **accuracy** |
| --- | --- | --- | --- | --- | --- |
| ≥ 23% | ≥1 | 93 (95%) | 65% | 66% | 66% |
|  | ≥5 | 75 (77%) | 59% | 71% | 65% |
|  | ≥10 | 53 (54%) | 47% | 78% | 63% |
|  | ≥20 | 38 (39%) | 46% | 86% | 66% |
|  | ≥50 | 27 (28%) | 60% | 100% | 80% |
|  | ≥100 | 17 (17%) | 67% | 100% | 84% |
| **HER2+CK+ tdEVs / total tdEVs** | **Total tdEVs** | **Patients**  ***N* (%)** | **sensitivity** | **specificity** | **accuracy** |
| ≥ 7% | ≥1 | 98 (100%) | 74% | 74% | 74% |
|  | ≥5 | 92 (94%) | 77% | 74% | 76% |
|  | ≥10 | 85 (87%) | 81% | 75% | 78% |
|  | ≥20 | 69 (70%) | 79% | 77% | 78% |
|  | ≥50 | 48 (49%) | 80% | 89% | 85% |
|  | ≥100 | 36 (37%) | 82% | 100% | 91% |
